# ANCO-GeneDB: annotations and comprehensive analysis of candidate genes for alcohol, nicotine, cocaine and opioid dependence

**DOI:** 10.1093/database/bay121

**Published:** 2018-11-06

**Authors:** Ruifeng Hu, Yulin Dai, Peilin Jia, Zhongming Zhao

**Affiliations:** 1 Center for Precision Health, School of Biomedical Informatics, The University of Texas Health Science Center at Houston, Houston, TX, USA; 2 Department of Psychiatry and Behavioral Sciences, The University of Texas Health Science Center at Houston, Houston, TX, USA; 3 Department of Biomedical Informatics, Vanderbilt University Medical Center, Nashville, TN, USA

## Abstract

Studies have shown that genetic factors play an important role in the risk to substance addiction and abuse. So far, various genetic and genomic studies have reported the related evidence. These rich, but highly heterogeneous, data provide us an unprecedented opportunity to systematically collect, curate and assess the genetic and genomic signals from published studies and to perform a comprehensive analysis of their features, functional roles and druggability. Such genetic data resources have been made available for other disease or phenotypes but not for major substance dependence yet. Here, we report comprehensive data collection and secondary analyses of four phenotypes of dependence: alcohol dependence, nicotine dependence, cocaine dependence and opioid dependence, collectively named as Alcohol, Nicotine, Cocaine and Opioid (ANCO) dependence. We built the ANCO-GeneDB, an ANCO-dependence-associated gene resource database. ANCO-GeneDB includes resources from genome-wide association studies and candidate gene-based studies, transcriptomic studies, methylation studies, literature mining and drug-target data, as well as the derived data such as spatial–temporal gene expression, promoters, enhancers and expression quantitative trait loci. All associated genes and genetic variants are well annotated by using the collected evidence. Based on the collected data, we performed integrative, secondary analyses to prioritize genes, pathways, eQTLs and tissues that are significantly enriched in ANCO-related phenotypes.

## Introduction

According to the definition by the American Society of Addiction Medicine and the latest edition of Diagnostic and Statistical Manual of Mental Disorders (DSM-V) (1), substance use disorders are a group of disorders resulting from the use of about 10 classes of drugs/substances, including alcohol, tobacco, marijuana, stimulants (e.g. cocaine) and opioids, among others. Substance use disorders occur when recurrent use of certain substances leads to clinically and functionally significant impairment, such as health problems, disability and failure to meet major responsibilities. Substance use disorders have become a major health, socioeconomical and behavioral issue across the world (2–4), causing 13% of all deaths worldwide and 9% of all disability-adjusted life years (5). Among these disorders, tobacco use disorder is the most common in the USA and nicotine sustaining tobacco smoking causes 1 in 10 deaths worldwide. Health consequences associated with smoking include circulatory disease, chronic obstructive pulmonary disease, hypertension, atherosclerosis and lung cancer (6, 7). Opioid drugs are another group of major substances causing deaths and expenditures. According to the statistics of the National Institute on Drug Abuse, the number of deaths involving opioid drugs had a 6-fold increase from 2002 to 2016, while death due to drug overdose has been increased 8-fold in the past two decades (8). The economic burden of opioid is increasing yearly, stemming from accidents, healthcare spending, lost productivity and incarceration. Accordingly, an opioid crisis was announced by the US government recently. Excessive alcohol use is the third leading cause of preventable death in the USA. Worldwide, excessive alcohol consumption accounts for 5.1% of the burden of disease and injury and 3.3 million deaths every year (9). Last but not the least, the use of cocaine has serious short- and long-term health effects. When cocaine is used in combination with alcohol, or other substances, the risk of damage increases greatly (10). Cocaine-taking could increase the level of dopamine, a natural chemical messenger that is associated with exercise and reward control in the brain circuitry. Accordingly, this substance use causes addiction and other adverse health consequences. The most frequent and serious health consequences of cocaine abuse include heart attacks and strokes, which can be fatal (11).

Substance use disorders affect both the physical and psychological well-being of individuals (12). Importantly, individuals with a substance use disorder are more likely to develop another substance use disorder or involve themselves in a new mental illness. For example, tobacco use disorders are prevalent in those individuals who consume alcohol and use other drugs, have attention deficit/hyperactivity disorder or are involved in addiction (13). Studies have found that genetic factors accounted for approximately half of the likelihood of an individual developing substance dependence (14, 15). Therefore, understanding the genetic basis and molecular consequences of substance use may contribute to the development of better treatment strategies. So far, there have been thousands of studies reporting various genetic and genomic evidence associated with substance use, including genome-wide association studies (GWASs), next-generation sequencing, gene expression, methylation, proteomics, metabolomics and therapeutic studies (16–20). These rich, yet highly heterogeneous, data provide us an unprecedented opportunity to systematically collect, curate and assess the genetic evidence from published studies and to perform a comprehensive analysis of their features, functional roles and druggability. Despite the high demand on searching substance-related data, there has been no dedicated database to curate genetic variants and genes related to substance use, let alone comprehensive feature analyses of the collected variants/genes. Like the databases in other complex diseases [e.g. Schizophrenia Gene Resource (SZGR) (21, 22) and Autism Gene Database (AutDB) (23)], systematic integration and curation of these discoveries with gene annotation and analyses could greatly help the investigators to filter, prioritize and clarify the risk factors underlying the etiology of substance use disorders.

In this study, we performed a comprehensive data collection as well as secondary analyses of genetic and genomic data related to four substances: alcohol, nicotine/tobacco, cocaine and opioid. Notably, according to DSM-V, substance abuse and substance dependence have been combined into single category of substance use disorders and the current names for the four phenotypes are Alcohol Use Disorder, Tobacco Use Disorder, Stimulant Use Disorder (including but not limited to cocaine) and Opioid Use Disorder. However, due to historical reasons, many studies use `dependence’ and/or `addiction’ when describing the phenotypes. To ensure the completeness of our database, we used alcohol dependence (AD), nicotine dependence (ND), cocaine dependence (CD) and opioid dependence (OD) to generally refer to these dependence-related phenotypes. We built the ANCO-GeneDB, an Alcohol, Nicotine, Cocaine and Opioid (ANCO) dependence-associated genetic resource that provides references for these substance dependence phenotypes.

## Data collection and implementation

**Figure 1:**
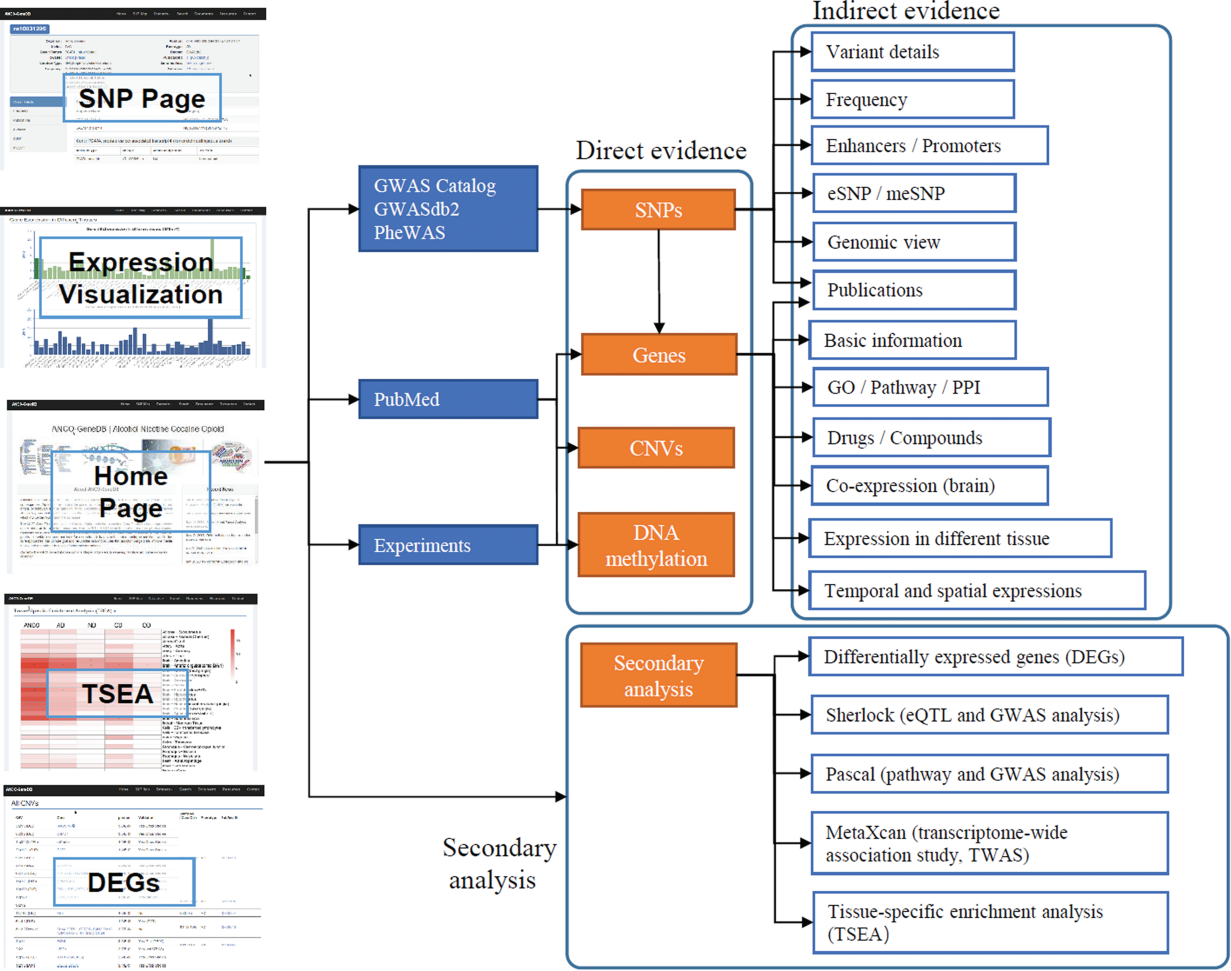
Schematic diagram describing data collection, features and applications of ANCO-GeneDB. The data in ANCO-GeneDB were collected and curated from various sources and were organized at three levels: (i) genes and genetic variants with direct association evidence in the ANCO phenotypes; (ii) indirect annotations on ANCO genes and genetic variants; and (iii) secondary analyses, including TSEA, Sherlock, PASCAL and MetaXcan analyses. Full names of the abbreviations are provided in the main text.

The data in ANCO-GeneDB were collected and curated from various sources (Figure 1), as summarized below and in the online document.

### Raw data search strategies

#### (1) Genetic variants

We collected genetic variants, including single nucleotide polymorphisms (SNPs) and short insertions/deletions, from GWAS Catalog (*P* < 1 × 10^−5^) (24), GWASdb2 (*P* < 1 × 10^−3^) (25) and Phenome-wide association studies (PheWAS) (*P* < 1 × 10^−3^) (26) databases. In each of these databases, we used a set of keywords to search for ANCO-related phenotypes. The keywords for AD included `alcohol dependence’, `alcohol consumption’, `alcohol craving’, `alcohol withdrawal’, `alcoholism’, `alcohol drinking’ and `alcohol use’. The keywords for ND included `nicotine dependence’, `alcohol and nicotine co-dependence’, `nicotine smoking’, `tobacco use’, `nicotine addiction’ and `nicotine use’. The keywords for the phenotype CD included `cocaine dependence’, `cocaine consumption’, `cocaine addiction’ and `cocaine use’. The keywords for the phenotype OD included `opioid dependence’, `opioid addiction’ and `opiate addiction’. Hereafter, we refer to these keywords as ANCO-dependence-related terms. If one SNP was reported in multiple studies, we kept all *P*-values for the SNP from these different studies and presented the data on the SNP page in ANCO-GeneDB.

#### (2) Genes

We curated ANCO-associated genes using two approaches: (i) genes defined by ANCO-associated genetic variants and (ii) genes that have been previously studied (literature search). For the former category of genes, we mapped the SNPs to genes following the dbSNP annotations (build 151). To collect the genes that have been previously studied in substance dependence or addiction, we developed a custom script to iteratively search for co-occurrence of each of the human protein-coding genes with ANCO dependence using the National Center for Biotechnology Information (NCBI) E-utilities functions. The generalized form of the query string was `<ANCO-dependence-related terms> [Title/Abstract] AND <gene symbol> [Title/Abstract]’, where `ANCO-dependence-related terms’ included the aforementioned keywords used in collection of genetic variants in step (i). The search results included those studies deposited into PubMed on or before 1 June 2018. Those genes that have been previously studied in ANCO-related phenotypes were recorded as well as the corresponding PubMed ID(s).

#### (3) DEGs

To obtain the differentially expressed genes (DEGs) between ANCO-dependence groups and control groups, we searched the NCBI Gene Expression Omnibus (GEO) using the aforementioned ANCO-dependence-related terms. We restricted the studies in `Homo sapiens’, as defined by the Organism item. GEO2R (https://www.ncbi.nlm.nih.gov/geo/geo2r/) was used to obtain the DEGs in each data set. To allow a comprehensive presentation of the data, we utilized two sets of criteria to define DEGs based on the adjusted *P*-values by the Benjamini and Hochberg (1995) method and fold change (FC): (i) adjusted *P* < 0.05 and |log_2_FC| > 1 and (ii) adjusted *P* < 0.2 and |log_2_FC| > 0.58.

#### (4) Drugs

DrugBank (Version 5.1.0) (27) and the Comparative Toxicogenomics Database (CTD) (Update 2017) (28) were retrieved for drugs and chemicals related to ANCO-associated genes from step (ii). For drugs from DrugBank, the drug type, the drug status and the role of the related gene information were collected. For drugs from CTD, the actions between the drug and its agent were recorded. In total, we collected 4788 DrugBank records and >777 000 CTD records involving 5221 ANCO-associated genes.

#### (5) CNVs

To collect ANCO-associated copy number variations (CNVs), we manually searched the PubMed by using the query string: `<copy number variation OR CNV> AND <ANCO-dependence-related term>’. We carefully reviewed each of the query results and manually extracted the CNVs that were significantly associated with ANCO phenotypes as well as the evidence from the literature.

#### (6) DNA methylation data

We collected DNA methylation data from two sources: (i) the AD-associated DNA methylation data in the prefrontal cortex region, including 1812 5’-C-phosphate-G-3’ sites (CpGs) identified in males and 154 CpGs in females, was acquired from the original lab (19) and (ii) other methylation data were obtained using the same method as we collected CNVs by manual curation from PubMed and online resources (29–33). In total, we collected 7471 CpGs from seven studies.

#### (7) GWAS summary statistics

We searched for GWAS summary statistics for ANCO-dependence phenotypes and obtained two data sets that were publicly available for ND and AD, respectively. The GWAS data for an ND-related trait, i.e. cigarettes per day, was from the Tobacco and Genetics Consortium. The study included 74 035 European ancestry individuals with imputed genotyping data for ∼2.5 million SNPs (34). The GWAS data for AD-related trait was from the Study of Addiction: Genetics and Environment (SAGE) (35). Samples were categorized using the DSM-IV criteria. The SAGE study included 2668 individuals (1235 cases and 1433 controls) of European ancestry genotyped with the Illumina 1 M BeadChip resulted in a set of ∼1 million SNPs (35).

### Annotation data

To facilitate functional studies and deep understanding of ANCO-dependence, we collected comprehensive information of functions, regulations and cross-domain correlations to annotate the curated genetic variants and genes, especially in disease-relevant tissues. For genetic variants, we provided the following annotations. (i) Expression quantitative trait loci (eQTL) in phenotype-relevant tissues. Many of the ANCO-associated genetic variants are located in non-coding regions and likely play regulatory roles. eQTLs are SNPs that are associated with gene expression, which can help find key SNPs for associated phenotype. We collected eQTL data in 13 brain tissues from Genotype-Tissue Expression (GTEx) Program (v7) (36) to annotate the genetic variants, because various brain regions have been recently reported to be involved in addiction-related phenotypes (37). (ii) Methylation quantitative trait loci (meQTL) in fetal brain (38). SNPs involved in meQTL, denoted as meSNP, were cross-linked with our ANCO-associated SNPs and the information was available to all SNPs, wherever applicable. (iii) Chromatin interaction annotations. We utilized the GWAS4D method (39, 40) to search for SNPs that were involved in chromatin interactions. If there were significant Hi-C interactions around an SNP, the annotation data would be available on the SNP detail information page. (iv) Enhancer and promoter annotations. Annotations for enhancers and promoters from the Roadmap Epigenomics Project were downloaded for 10 brain samples (41). We mapped our ANCO-associated SNPs to these enhancers and promoters, with a flanking region of 5000 base pairs upstream or downstream of each SNP. Such annotations are dynamically presented on the ANCO-GeneDB website. (v) General annotations are available to all SNPs, including SNP coordinates (in both Genome Reference Consortium Human Build 37 (GRCh37) and GRCh38) and contexts, population frequency in various cohorts (e.g. the 1000 Genomes Project, TOPMED and TWINSUK), variant types (SNPs versus short insertions/deletions) and function categories of genes (e.g. intronic versus amino acid changing).

For ANCO-associated genes, we provide the following annotations. First, gene expression across multiple tissues from two sources: GTEx (v7) (47 tissues, each with ≥30 samples) and ENCODE (44 tissues, each with ≥2 samples) (42). The average Reads Per Kilobase (kb) of transcript per Million mapped reads values of each gene in multiple tissues were presented through bar plots on the gene page. Second, gene co-expression data. For each ANCO-associated gene, we measured its co-expression relationship with other genes in the transcriptome using the GTEx (v7) multiple tissue expression data. To make it visible, it displays the 10 most positively co-expressed genes and the 10 most negatively co-expressed genes with each query gene in brain frontal cortex (BA9), ordered by Pearson Correlation Coefficient. Third, BrainSpan temporal–spatial expression data in four brain regions (sub-cortical regions, sensory-motor regions, frontal cortex and temporal–parietal cortex) and three developmental time periods [Stage 1: fetal (13–26 postconceptional weeks), Stage 2: early infancy to late childhood (4 months–11 years) and Stage 3: adolescence to adulthood (13–23 years)] (43, 44). Lastly, general annotations, including Gene Ontology (GO), pathway (including KEGG and Reactome), protein–protein interaction (PPI, STRING v10.5) (45), drug targets and publications, wherever available.

### Secondary analyses

To better serve the research community of addiction, we conducted a series of advanced analyses, providing cross-domain evidence for gene prioritization. These analyses included Tissue-Specific Enrichment Analysis (TSEA) (v1.0) aiming to identify the phenotype-related tissues for each trait (46), enrichment analysis of tissue-specific eQTL using eQTLEnrich (v1_062718) (47, 48), integrative analysis of eQTL and GWAS data using Sherlock (accessed 15 May 2018) (49), gene- and pathway-based integrative analysis using PASCAL (accessed 15 May 2018) (50, 51) and Transcriptome-Wide Association Study (TWAS) using MetaXcan (accessed 22 May 2018) (52).

#### (1) TSEA

To identify the specific tissues in which the ANCO-associated genes were mostly enriched, we applied TSEA method to the genes that were obtained by GWAS SNPs and genes that were obtained from literature search, respectively. TSEA is our in-house tool that implements Fisher’s Exact Test to assess whether a list of query genes are enriched in tissues based on GTEx (v7) (47 tissues, each with ≥ 30 samples) reference pan-tissue panel.

#### (2) Enrichment analysis of tissue-specific eQTLs

eQTLEnrich tool is designed to test the distribution of GWAS *P*-values for each set of eQTLs (False discovery rate (FDR) < 0.05) in trait associations compared to an empirical null distribution sampled from non-significant variant gene-expression associations (FDR > 0.05) (48). For each tissue, to obtain the fold enrichment, the fraction of eQTLs (FDR < 0.05) with GWAS variants (*P* < 0.05) is compared to the expectation 5%, assuming that GWAS *P*-values are in uniform distribution. To calculate the adjusted fold enrichment, the fold enrichment for each tissue–trait pair is divided by the fold enrichment of all null eQTL SNPs (eSNPs) (GWAS *P* < 0.05) for the tissue–trait pair. Thus, eQTLEnrich can be used to evaluate the impact (enrichment) of a set of moderately significant GWAS SNPs in tissue–trait pairs. We used GWAS summary statistics of AD and ND as the input and conducted eQTLEnrich for each of the 44 tissues with pre-trained eQTL-database (GTEx v6) in European ancestry population.

#### (3) Gene and pathway scoring

To obtain an overall assessment of genes based on GWAS summary statistics, we applied PASCAL, a method that combines multiple SNPs mapped to genes and provides gene-based *P*-values, to ND and AD GWAS data, respectively. PASCAL uses SNP *P*-values from GWA studies and corrects for linkage disequilibrium structure. We used the default setting of PASCAL, including an upstream and downstream window of 50 kb pairs per gene, minor allele frequency > 0.05 and the reference panel of European from the 1000 Genomes Project.

#### (4) Integrative analysis of eQTL and GWAS data

As many disease-associated SNPs are located in non-coding regions with potential regulatory roles, we applied Sherlock to integrate eQTL and GWAS data to assess the load of combined information from the two lines of evidence. Sherlock uses a Bayesian statistical method to match the signature of genes from eQTL in each tissue or cell type with patterns of association in GWAS data set. Because Sherlock does not apply the stringent genome-wide significance level (such as 5 × 10^−8^), it has the advantage to evaluate SNPs with weak to moderate association evidence. Accordingly, it provides more chance to discover functionally important genes. Due to the data availability, we applied Sherlock to only ND and AD GWAS data sets by using the GTEx (v7) eQTL data from each of the 47 tissues.

#### (5) MetaXcan

To conduct TWAS, we applied MetaXcan using each of the 47 human tissues available from GTEx (v7). TWAS estimates the genetically regulated expression (GReX) based on proximate SNPs with pre-trained weights and assesses the difference of GReX in trait samples and control samples.

**Figure 2:**
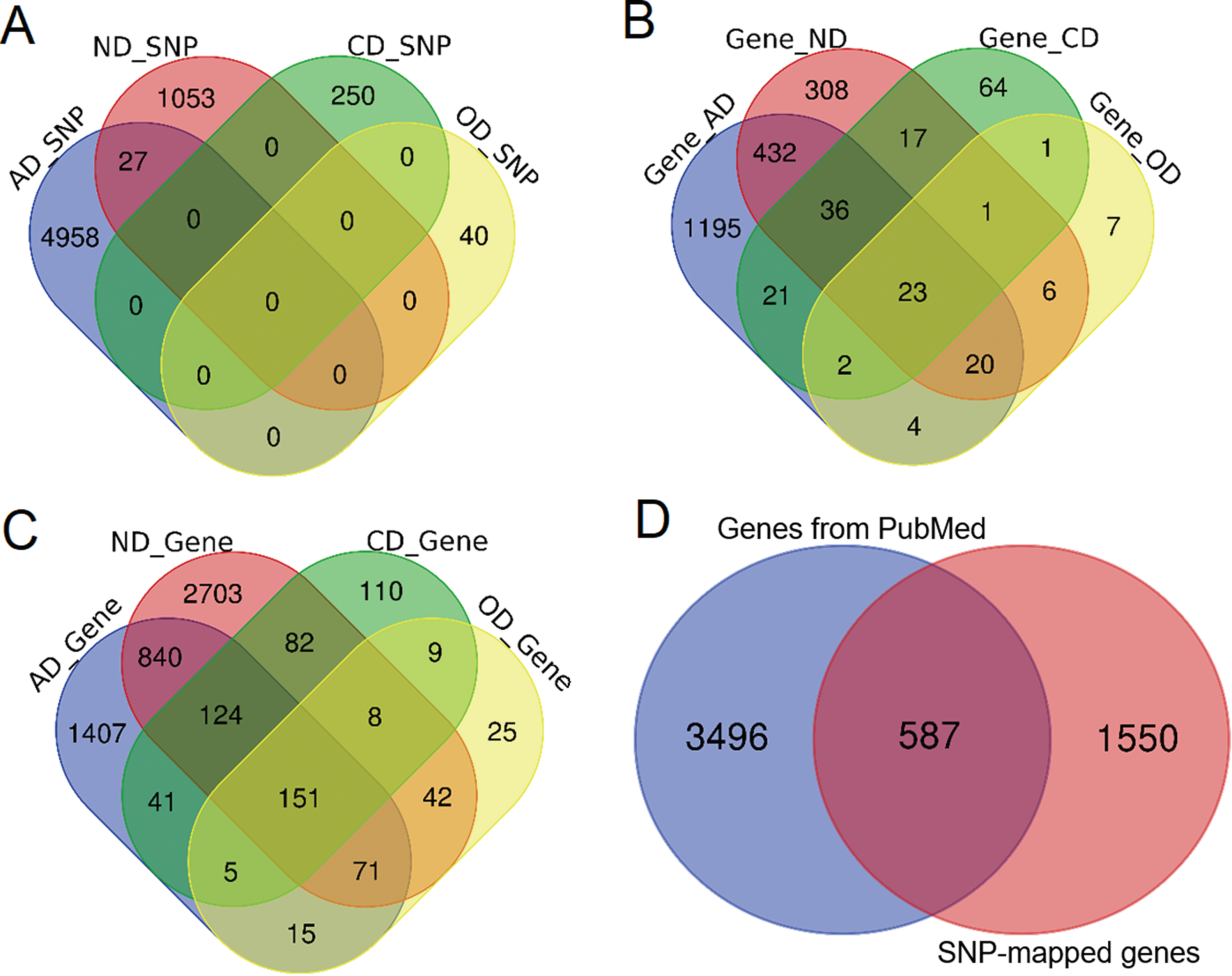
Comparison of genetic variants and genes. **(A)** Venn diagram comparing the genetic variants associated with each phenotype. **(B)** Venn diagram comparing ANCO genes (all genes obtained from two approaches). **(C)** Venn diagram comparing SNP-mapped genes. **(D)** Venn diagram comparing genes from two sources (SNP-mapped genes and genes extracted from the literature).

### Database structure

All the collected data and secondary analysis results are organized and indexed in a MySQL database. The ANCO-GeneDB website is implemented on a Linux server using PHP. Open-source JavaScript framework is employed to display tables and figures. We will routinely update the database every quarter of the year to include ANCO-related data from up-to-date studies. Our database design also allows us to add more substance use disorders in the future.

## Results

The data in ANCO-GeneDB is organized at three levels (Figure 1). (i) Genetic variants and genes with direct association evidence with the ANCO phenotypes. Such evidence is typically obtained by comparing a group of patient samples and a group of normal samples, so that an association test can be performed to identify the variants or genes with statistically significant difference between the two groups. Examples include GWAS and candidate gene studies, transcriptomic studies reporting DEGs, methylation studies reporting differentially methylated probes or regions and literature mining. (ii) Indirect information for ANCO-associated genetic variants and genes, especially in disease-relevant tissues. Such data include drug-target annotations, brain spatiotemporal gene expression profiles, brain promoter and enhancer annotations and eQTL. (iii) Integrative results from secondary analyses, including enriched pathways, top-ranked genes by tissues and disease-relevant brain regions. All the results from the secondary analyses are available in our online database. Therefore, ANCO-GeneDB provides not only the data from the original studies but also the data from advanced analyses to the research community, providing a one-stop shop of genetic variants and genes associated with the four major substance use disorders.

**Table 1:** Data summary of ANCO-GeneDB

**Data set**	**Number**				
	**AD**	**ND**	**CD**	**OD**	**ANCO**
SNPs	4985	1080	250	40	6328
Genes (SNP-mapped^*^)	2654 (1733^*^)	4021 (843^*^)	530 (165^*^)	326 (64^*^)	5633(2137^*^)
GEO data sets	4	3	2	0	9
Drugs (DrugBank)	3371	4675	2243	1969	4788
CNVs	17	1	1	10	29
Enhancers					510 567 (within 5 kb)
Promoters					71 325 (within 5 kb)
meDNA					7471 CpGs
eSNPs					804 (in brain tissues)
meSNPs					396 (in fetal brain tissues)

^*^The number of genes mapped by the significant SNPs.

### Data statistics

Based on our collected data and analysis, we curated a total of 6328 SNPs from three resources for the four phenotypes, including 4985, 1080, 250 and 40 SNPs for AD, ND, CD and OD, respectively. A direct comparison did not show much overlap among these SNPs, although 27 SNPs were shared between AD and ND (Figure 2A). In addition, 396 SNPs had been identified as meSNPs in the meQTL data from fetal brain, and 804 SNPs were eSNPs from GTEx eQTL data in frontal cortex. A total of 36 SNPs were found with significant Hi-C interactions. There were >510 000 enhancers and 71 000 promoters located within 5 kb upstream or downstream of these SNPs in 10 brain cell lines. Using the default annotation by dbSNP, we obtained 2137 ANCO-associated genes (Figure 2B). From literature search, we identified 4083 genes that had been previously studied in ANCO-related phenotypes from 62 000 studies (Figure 2C). Among them, *ALDH2*, *EGFR*, *BDNF* and *CAMP* are the most frequently studied genes for AD, ND, CD and OD, respectively. There are 587 genes shared between the two resources, resulting in 5633 unique ANCO-associated genes (Figure 2D). All the genes can be examined by the tissue-specific expression from our website in the gene detail page. For DEGs, after manually reading through and carefully checking each of the GEO data set, we obtained 12 valid data sets that were appropriate for our detection of DEGs associated with ANCO dependences, i.e. each with ≥3 cases and controls. A total of 29 CNV regions were identified, mapped and curated in our database. Table 1 shows the data summary of our database.

**Figure 3:**
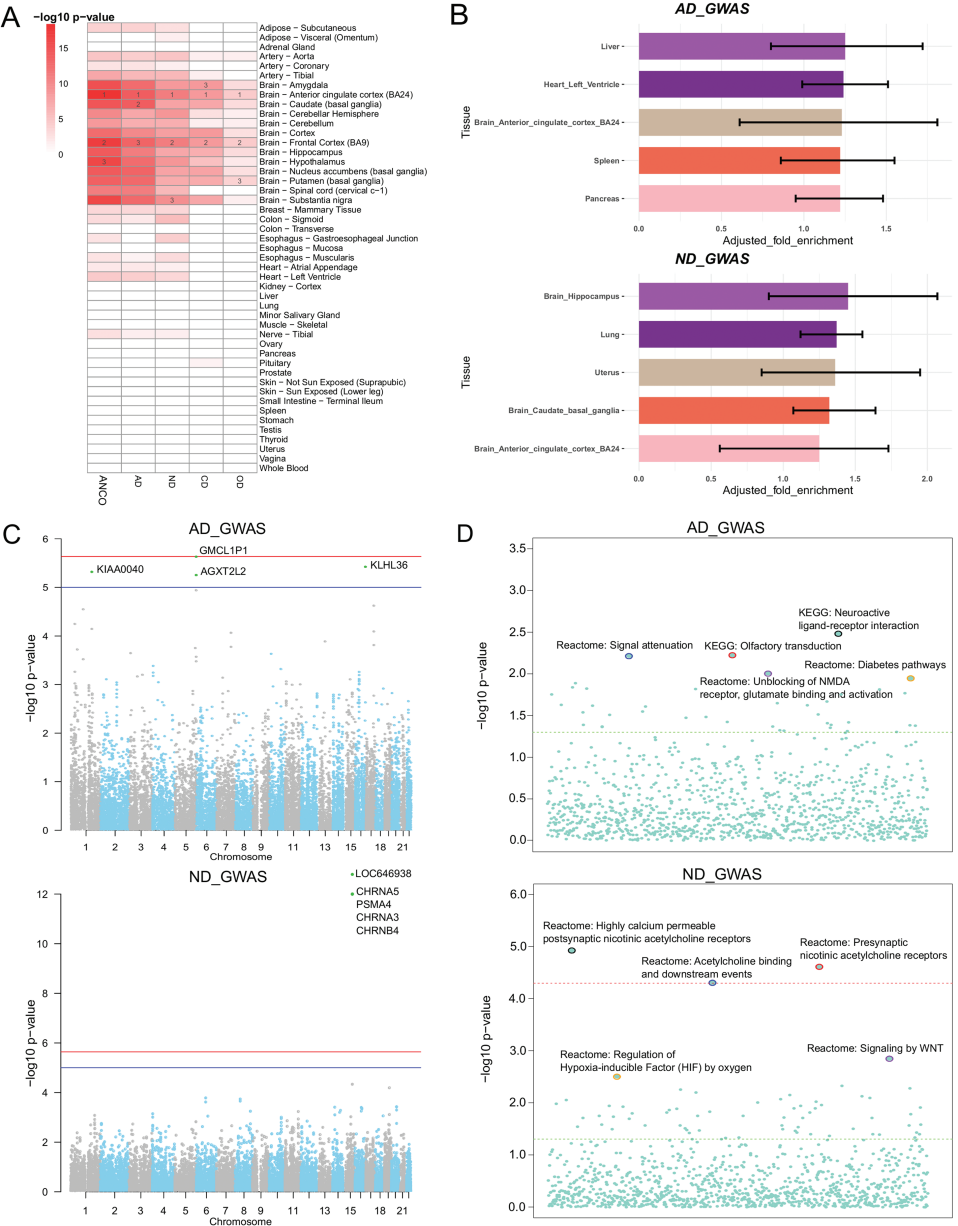
Secondary analysis of ANCO-associated genetic variants and genes. **(A)** TSEA of ANCO-associated genes (each individual gene set and the combined) using 47 GTEx (v7) tissues. Color is proportional to –log10 (*P*-value), where *P*-value was obtained from Fisher’s Exact Test. For each gene set, the top 3 most significantly enriched tissues are labeled. **(B)** eQTLEnrich analysis of the GWAS summary statistics for AD and ND (GTEx v6). The x-axis shows the adjusted fold enrichment. The black lines display 95% confidence interval. **(C)** Manhattan plots showing the distribution of gene-based *P*-values obtained by PASCAL analysis. The blue line indicates *P* = 1 × 10^−5^ and red line for *P* = 2.31 × 10^−6^ (Bonferroni correction threshold). **(D)** Distribution of the pathway enrichment analysis by PASCAL. The green line indicates *P* = 0.05 and red line for *P* = 4.64 × 10^−5^ (Bonferroni correction threshold).

**Figure 4:**
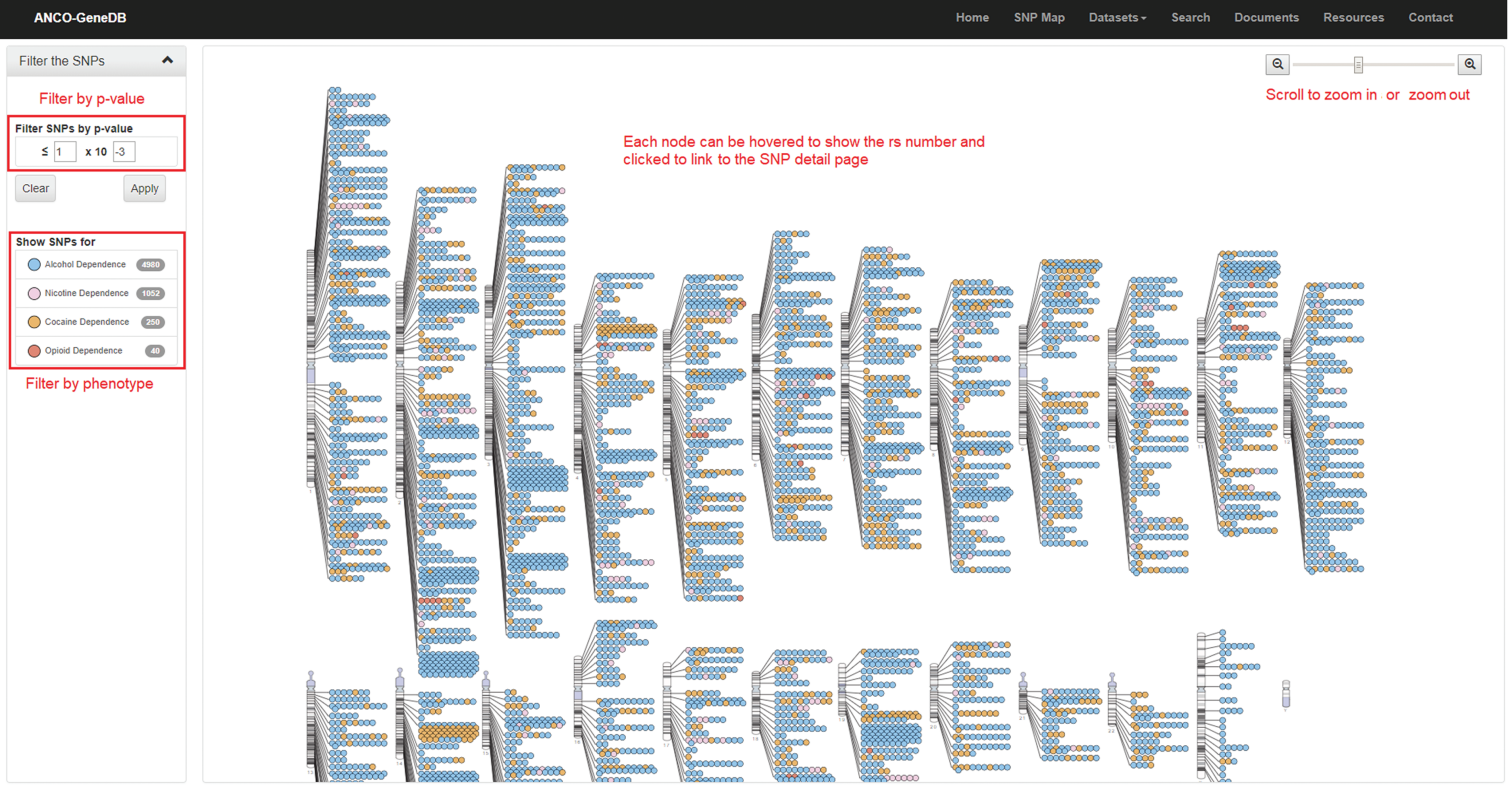
The SNP Map page. A genome-wide display of all the collected SNPs for ANCO phenotypes. Each node indicates an SNP and node color represents the phenotype. The user can click each SNP node linking to the detailed SNP information page and filter out the data based on *P*-value cut-off or phenotype (Note: the display of SNPs on chromosomes is adapted from the GWAS Catalog).

### Secondary data analyses

We applied TSEA to ANCO-associated genes identified from GWAS and the literature, respectively. As a result, we found that the SNP-mapped genes were significantly enriched in multiple brain regions (Figure 3A; Supplementary Table S1). Specifically, anterior cingulate cortex was the most significantly enriched tissue in all five sets of genes (*P* = 6.80 × 10^−15^ for AD; *P* = 4.95 × 10^−12^ for ND; *P* = 1.63 × 10^−9^ for CD; *P* = 2.37 × 10^−5^ for OD; and *P* = 3.79 × 10^−19^ for ANCO). Frontal cortex (BA9) was the second most significantly enriched tissue in four sets (*P* = 4.95 × 10^−12^ for ND; *P* = 1.63 × 10^−9^ for CD; *P* = 2.37 × 10^−5^ for OD; and *P* = 6.46 × 10^−18^ for ANCO) and the third one in AD (*P* = 4.16 × 10^−14^ for AD). Other regions of interest included Amygdala (*P* = 2.43 × 10^−13^ for AD; *P* = 2.53 × 10^−9^ for ND; *P* = 2.18 × 10^−9^ for CD; *P* = 5.40 × 10^−4^ for OD; and *P* = 6.06 × 10^−16^ for ANCO), caudate (basal ganglia) and hypothalamus, putamen (basal ganglia) and substantia nigra. These results further confirm that different brain regions are likely involved in the etiology of substance use dependence.

Several GWA studies have been conducted for addiction-related traits. However, most of the significant SNPs identified at the stringent genome-wide significance level fell in non-coding regions with limited annotations, had no obvious functional consequences and only accounted for a small part of the heritability of disease. To further interpret the GWAS results, we leveraged the whole-genome summary statistics with multilevel bioinformatics tools to investigate the data from distinct yet complementary aspects. First, our tissue-specific enrichment test of eQTL data showed that eQTLs in liver and brain hippocampus were most enriched with GWAS signals for AD (*P* = 2.38 × 10^−2^) and ND (*P* = 1.70 × 10^−2^), respectively (Figure 3B; Supplementary Table S2). Second, by assessing the combined information at the gene level, we found a cluster of genes, including *CHRNA5, PSMA4, CHRNA3* and *CHRNB4* on chromosome 15, were the most significantly associated genes with ND, consistent with the original GWAS study (34) (Figure 3C). At the pathway level, the three most significant pathways for ND were the pathway of highly calcium permeable postsynaptic nicotinic acetylcholine receptors (*P* = 1.14 × 10^−5^), presynaptic nicotinic acetylcholine receptors (*P* = 2.35 × 10^−5^) and acetylcholine binding and downstream events (*P* = 4.77 × 10^−5^; Figure 3D). And the top three ranked pathways for AD were neuroactive ligand receptor interaction (*P* = 3.22 × 10^−3^), olfactory transduction (*P* = 5.84 × 10^−3^) and signal attenuation (*P* = 5.99 × 10^−3^).

Third, we combined GWAS and eQTL data using Sherlock in a tissue-specific way, aiming to find consistent evidence from the two domains. We particularly examined the results from 13 brain regions and pituitary, as these regions and tissues were presumably most relevant to addiction-related traits. As shown in Supplementary Table S3, several genes were found to be recurrently significant in multiple regions, such as *CHRNA5*, *ADAM17*, *CYP2A7* and *WDR18*. Interestingly, the gene *CHRNA5* was the most significant gene in four regions (amygdala, anterior cingulate cortex BA24, cerebellar hemisphere and hypothalamus) and the second most significant gene in cerebellum and hippocampus. For AD, we also found several recurrent genes among the top 10 most significant genes, such as *HLA-C* (major histocompatibility complex, class I, C), *POLE* (DNA polymerase epsilon, catalytic subunit) and *TDRD6* (tudor domain containing 6), among others. Notably, the HLA class I gene *HLA-C* (ranked top 10 in eight regions) is located in the major histocompatibility complex region on chromosome 6. This region had been repeatedly reported to be associated with several mental disorders such as schizophrenia (53–55), implying that there might be shared genetic contribution between AD and other mental disorders. Lastly, we applied MetaXcan to prioritize genes with significant GReX in ND and AD cohorts (Supplementary Table S4). *CHRNA5* was again found within top-ranked genes in 10 out of the 14 brain-related tissues. We made the Manhattan plots (56) available on our website to all tissues for both Sherlock and MetaXcan results to show the distributions of tissue-specific significant genes. Collectively, we explored ANCO-associated genetic variants and genes in multiple trait-relevant tissues for their potential functions through various methods. These systematic analyses provided insights into better interpretation of the GWAS data.

### Web interface

We launched a user-friendly website to facilitate the use of our ANCO-GeneDB. The website allows users with full access to all the curated data and analysis results as mentioned above, including ANCO-associated genetic variants and genes and their annotations, secondary analysis results and resource links. We implemented scripts to enable dynamic data visualization and various functions to explore the data, including browsing, searching, sorting and conditional selecting, among others. A header bar was available on all pages of the website, so users can access and jump to any data set from any page on the website.

We organize our data by their data types, resulting in five major groups that are accessible from the `Datasets’ button: genes, SNPs, Drugs, CNVs and meDNA. In the table view of each data set, to reduce the loading time, we organize the data by multiple pages wherever applicable, with 25 entries per page by default. Users can easily change the number of entries per page by using the pop-up selection menu we provide. In each data set table, sorting is made available through a single click on the column headers, enabling either ascending or descending orders. A fuzzy search function is also available in each data set table, facilitating users to search the content of table cells. In addition, a universal search page is available to enable the advanced searching function using Entrez ID, gene symbol, SNP rsID, phenotype and chromosome location.

As one example, we build an SNP Map web page (Figure 4) to show the locations of collected SNPs on each chromosome. On this page, users can filter the SNPs by *P*-value and phenotype. SNPs are labeled in different colors by phenotype. Each SNP node on this page can be clicked and then linked to the detailed SNP page, which is the major information page for ANCO-associated SNPs. On the SNP web page, a comprehensive list of annotations is displayed, including the basic information about the SNP (alleles, associated genes, variant type, etc.), minor allele frequencies in different populations, related publications, GWAS *P*-values in different studies, eSNP annotation, meSNP annotation, genomic view and enhancers and promoters nearby the SNP. Similarly, a gene page presents detailed information for each ANCO-associated gene. Specifically, each gene page consists of various annotation data, including the basic information about the gene, methylation annotation, multiple tissue expression data from two panels (GTEx v7 and ENCODE), temporal–spatial expression in four different brain regions and three developmental stages, co-expressed genes (both positively and negatively) in brain frontal cortex (GTEx v7), related drugs/compounds, GO/pathway/PPI annotations and related publications.

## Conclusion

ANCO-GeneDB is a comprehensive genetic and genomic database for AD, ND, CD and OD. All the curated data are publicly accessible. Users can browse, retrieve and analyze the genes and their features for AD, ND, CD and OD individually and cross-phenotype. Substance addiction research is currently under rapid growth, and more genetic data sets and findings will be reported in the near future. We will routinely update the data for better understanding of addiction mechanisms, progression, neurobiology and identification of drug-targetable alterations. Our long-term goal is to have this database as a hub for ANCO-related genes and features and expand it to other substance use disorder when such data become available.

## Supplementary Material

Supplementary Data

## Notes

**Database URL:** https://bioinfo.uth.edu/ancogenedb/
