# Supplementary material for: ANCO-GeneDB: annotations and comprehensive analysis of candidate genes for alcohol, nicotine, cocaine and opioid dependence
Source: Database (Oxford). 2018 Nov 6;2018:bay121. doi: 10.1093/database/bay121 (PMC6310508; doi:10.1093/database/bay121)
Supplement: Supplementary Data [file anco_db_supplementary_tables.pdf]

Table S1. Results (p values) of Tissue-Specific Enrichment Analysis (TSEA)

Tissue	P value				
	ANCO	AD	ND	CD	OD
Adipose - Subcutaneous	0.000155922	0.000155922	0.001298167	0.163027126	0.163027126
Adipose - Visceral (Omentum)	0.115317091	0.07434769	0.029026166	0.265736548	0.203783908
Adrenal Gland	0.115669911	0.115669911	0.553591292	0.115669911	0.793555289
Artery - Aorta	1.34E-05	6.17E-05	1.34E-05	0.023709219	0.024878251
Artery - Coronary	0.004112353	0.004112353	0.015174088	0.052215022	0.163027126
Artery - Tibial	3.08E-08	5.88E-07	5.88E-07	0.023709219	0.068618715
Brain - Amygdala	6.06E-16	2.43E-13	2.53E-09	2.18E-09	0.000539975
Brain - Anterior cingulate cortex (BA24)	3.79E-19	6.80E-15	4.95E-12	1.63E-09	2.37E-05
Brain - Caudate (basal ganglia)	1.46E-15	6.80E-15	8.04E-09	3.94E-08	0.000539975
Brain - Cerebellar Hemisphere	5.60E-11	2.12E-08	8.52E-10	0.001390792	0.024878251
Brain - Cerebellum	2.07E-09	1.25E-07	2.17E-07	0.023709219	0.024878251
Brain - Cortex	2.34E-13	8.24E-11	6.03E-09	2.18E-09	0.002186178
Brain - Frontal Cortex (BA9)	6.46E-18	4.16E-14	4.95E-12	1.63E-09	2.37E-05
Brain - Hippocampus	3.49E-15	7.27E-12	1.88E-08	1.79E-07	0.000539975
Brain - Hypothalamus	4.09E-17	2.43E-13	1.40E-09	3.13E-06	0.00786015
Brain - Nucleus accumbens (basal ganglia)	3.49E-15	4.16E-14	1.41E-08	1.10E-08	0.002186178
Brain - Putamen (basal ganglia)	1.93E-14	1.01E-13	7.33E-08	5.25E-08	0.000119326
Brain - Spinal cord (cervical c-1)	7.27E-12	7.27E-12	1.01E-06	0.052215022	0.068618715
Brain - Substantia nigra	4.09E-17	1.01E-13	2.27E-10	3.94E-08	0.024878251
Breast - Mammary Tissue	0.000348745	0.000458995	0.000348745	0.163027126	0.163027126
Colon - Sigmoid	0.000819439	0.009038065	1.42E-06	0.329450374	0.329450374
Colon - Transverse	0.82179487	0.82179487	0.82179487	0.82179487	0.82179487
Esophagus - Gastroesophageal Junction	0.004169941	0.066705152	5.36E-05	0.512285369	0.559549141
Esophagus - Mucosa	0.995270545	0.995270545	0.995270545	0.995270545	0.995270545
Esophagus - Muscularis	0.002826265	0.036299088	0.00069749	0.575084581	0.575084581
Heart - Atrial Appendage	0.009239334	0.009239334	0.015174088	0.265736548	0.203783908
Heart - Left Ventricle	3.61E-05	5.41E-05	0.000232497	0.052215022	0.329450374
Kidney - Cortex	0.949201912	0.949201912	0.949201912	0.949201912	0.949201912
Liver	0.914577681	0.914577681	0.416374554	0.407567816	0.407567816
Lung	0.663755599	0.663755599	0.663755599	0.663755599	0.663755599
Minor Salivary Gland	0.135582044	0.135582044	0.255580942	1	1

Muscle - Skeletal	0.080313216	0.080313216	0.193811234	0.332170686	0.793555289
Nerve - Tibial	0.001638878	0.026201627	0.022761132	0.332170686	0.793555289
Ovary	0.966014589	0.966014589	0.966014589	0.966014589	0.966014589
Pancreas	0.998282028	0.998282028	0.998282028	0.998282028	0.998282028
Pituitary	0.901731754	0.914577681	0.901731754	0.03974154	0.901731754
Prostate	0.680167506	0.680167506	0.949201912	0.949201912	0.949201912
Skin - Not Sun Exposed (Suprapubic)	1	1	1	1	1
Skin - Sun Exposed (Lower leg)	0.640378	0.640378	0.959044014	0.959044014	0.959044014
Small Intestine - Terminal Ileum	0.999682015	0.999682015	0.999682015	0.999682015	0.999682015
Spleen	0.949201912	0.949201912	0.949201912	0.949201912	0.949201912
Stomach	0.699436427	0.699436427	0.699436427	0.94721774	0.699436427
Testis	1	1	1	1	1
Thyroid	0.329768045	0.265437048	0.329768045	0.94721774	0.699436427
Uterus	0.181000185	0.181000185	0.312280915	0.181000185	0.329450374
Vagina	0.949201912	0.949201912	0.949201912	0.949201912	0.949201912
Whole Blood	0.793555289	0.793555289	0.793555289	0.793555289	0.793555289

Table S2. Results of eQTLenrich analysis for AD-GWAS dataset and ND-GWAS dataset													
**GWAS_Trait**	**Tissue_eQTL**	**Number_best_eQTL_per_eGene_variants_overlap_with_GWAS**	**Number_null_variants_overlap_with_GWAS**	**OBS_number_eQTLs_with_GWAS_p<0.05**	**EXP_number_eQTLs_with_GWAS_p<0.05**	**Number_true_positive_trait_associations_amongst_eQTLs_with_GWAS_p<0.05_(OBS-EXP)**	**Fold-Enrichment_(OBS/EXP)**	**EXP_number_eQTLs_with_GWAS_p<0.05_Adj**	**Adjusted_number_true_positive_trait_associations_amongst_eQTLs_with_GWAS_p<0.05_(Adj_OBS-EXP)**	**Adjusted_Fold-Enrichment_(Adj_OBS/EXP)**	**Lower_bound_95%_confidence_interval**	**Upper_bound_95%_confidence_interval**	**Enrichment_P-value_(matched_on_confounders)**
ND	Adipose_Subcutaneous	2367	2834	143	118	25	1.21	115	28	1.24	1.1	1.41	1.03E-01
ND	Adipose_Visceral_Omentum	1249	4016	72	62	10	1.15	76	0	0.95	0.78	1.13	5.31E-01
ND	Adrenal_Gland	943	4452	50	47	3	1.06	55	0	0.91	0.72	1.09	4.78E-01
ND	Artery_Aorta	1833	3372	88	92	-4	0.96	103	0	0.85	0.73	0.98	9.31E-01
ND	Artery_Coronary	672	4905	34	34	0	1.01	33	1	1.03	0.71	1.31	1.50E-01
ND	Artery_Tibial	2289	2646	115	114	1	1	112	3	1.02	0.89	1.16	2.07E-01
ND	Brain_Anterior_cingulate_cortex_BA24	317	5244	20	16	4	1.26	16	4	1.25	0.56	1.73	4.60E-04
ND	Brain_Caudate_basal_ganglia	703	5010	49	35	14	1.39	37	12	1.32	1.07	1.64	7.98E-01
ND	Brain_Cerebellar_Hemisphere	904	5014	54	45	9	1.19	50	4	1.09	0.83	1.33	3.05E-02
ND	Brain_Cerebellum	1230	4664	76	62	15	1.24	65	11	1.17	0.96	1.43	3.12E-01
ND	Brain_Cortex	756	5054	41	38	3	1.08	42	0	0.97	0.75	1.22	7.99E-01
ND	Brain_Frontal_Cortex_BA9	582	5048	38	29	9	1.31	31	7	1.21	0.9	1.59	3.25E-01
ND	Brain_Hippocampus	322	5419	24	16	8	1.49	17	7	1.45	0.9	2.07	1.70E-02
ND	Brain_Hypothalamus	330	5455	15	17	-2	0.91	18	0	0.85	0.46	1.19	3.17E-01
ND	Brain_Nucleus_accumbens_basal_ganglia	588	5121	39	29	10	1.33	33	6	1.18	0.93	1.5	4.52E-01
ND	Brain_Putamen_basal_ganglia	427	5063	28	21	7	1.31	23	5	1.22	0.83	1.7	2.88E-01
ND	Breast_Mammary_Tissue	1187	4421	72	59	13	1.21	59	13	1.23	0.98	1.46	1.13E-03
ND	Cells_EBV-transformed_lymphocytes	843	4077	50	42	8	1.19	51	0	0.99	0.79	1.22	7.95E-01
ND	Cells_Transformed_fibroblasts	2502	2274	149	125	24	1.19	129	20	1.16	1.02	1.31	1.35E-03
ND	Colon_Sigmoid	812	4587	41	41	0	1.01	47	0	0.87	0.68	1.08	9.75E-01
ND	Colon_Transverse	1324	4203	68	66	2	1.03	72	0	0.94	0.77	1.11	4.16E-01
ND	Esophagus_Gastroesophageal_Junction	793	4554	45	40	5	1.13	39	6	1.15	0.89	1.44	4.26E-01
ND	Esophagus_Mucosa	2069	3184	127	103	24	1.23	112	15	1.14	0.99	1.27	1.30E-04
ND	Esophagus_Muscularis	1954	3197	112	98	14	1.15	101	11	1.1	0.96	1.27	3.65E-01
ND	Heart_Atrial_Appendage	1125	4019	62	56	6	1.1	62	0	1	0.82	1.19	8.44E-01

ND	Heart_Left_Ventricle	1309	3504	82	65	17	1.25	67	15	1.23	0.98	1.45	<1.00e-05
ND	Liver	441	4646	28	22	6	1.27	24	4	1.17	0.85	1.66	8.93E-01
ND	Lung	2068	3444	141	103	38	1.36	103	38	1.37	1.12	1.55	<1.00e-05
ND	Muscle_Skeletal	1947	2490	102	97	5	1.05	99	3	1.03	0.9	1.18	8.68E-01
ND	Nerve_Tibial	2878	2957	160	144	16	1.11	146	14	1.1	0.97	1.22	3.20E-02
ND	Ovary	442	5344	26	22	4	1.18	23	3	1.15	0.78	1.56	4.23E-01
ND	Pancreas	1266	3924	67	63	4	1.06	69	0	0.97	0.8	1.15	7.33E-01
ND	Pituitary	619	5581	36	31	5	1.16	31	5	1.16	0.9	1.54	8.82E-01
ND	Prostate	397	5729	21	20	1	1.06	21	0	1	0.7	1.43	9.42E-01
ND	Skin_Not_Sun_Exposed_Suprapubic	1534	3906	81	77	4	1.06	88	0	0.92	0.78	1.07	7.17E-01
ND	Skin_Sun_Exposed_Lower_leg	2501	3025	137	125	12	1.1	136	1	1.01	0.9	1.14	8.95E-01
ND	Small_Intestine_Terminal_Ileum	357	5800	21	18	3	1.18	18	3	1.16	0.74	1.69	3.12E-01
ND	Spleen	757	5039	36	38	-2	0.95	39	0	0.93	0.73	1.21	9.98E-01
ND	Stomach	1036	4281	59	52	7	1.14	52	7	1.14	0.87	1.36	1.64E-02
ND	Testis	2696	5368	134	135	-1	0.99	155	0	0.87	0.77	0.98	9.99E-01
ND	Thyroid	2805	2895	183	140	43	1.3	155	28	1.18	1.05	1.31	1.62E-03
ND	Uterus	267	5648	19	13	6	1.42	14	5	1.36	0.85	1.95	2.14E-01
ND	Vagina	218	5872	11	11	0	1.01	12	0	0.95	0.54	1.53	8.86E-01
ND	Whole_Blood	1851	2497	114	93	21	1.23	96	18	1.19	1.04	1.36	9.13E-02
AD	Adipose_Subcutaneous	1301	1487	83	65	18	1.28	79	4	1.05	0.9	1.24	3.81E-01
AD	Adipose_Visceral_Omentum	678	2042	38	34	4	1.12	47	0	0.81	0.67	0.98	1.00E+00
AD	Adrenal_Gland	502	2295	34	25	9	1.35	31	3	1.1	0.88	1.39	6.73E-01
AD	Artery_Aorta	988	1769	65	49	16	1.32	59	6	1.11	0.92	1.32	5.07E-01
AD	Artery_Coronary	360	2549	20	18	2	1.11	21	0	0.95	0.65	1.27	7.59E-01
AD	Artery_Tibial	1322	1460	87	66	21	1.32	76	11	1.14	0.95	1.34	9.80E-04
AD	Brain_Anterior_cingulate_cortex_BA24	159	2743	13	8	5	1.64	11	2	1.23	0.61	1.81	2.58E-02
AD	Brain_Caudate_basal_ganglia	389	2672	26	19	7	1.34	24	2	1.07	0.83	1.47	9.96E-01
AD	Brain_Cerebellar_Hemisphere	507	2625	30	25	5	1.18	34	0	0.89	0.69	1.11	9.44E-01
AD	Brain_Cerebellum	672	2465	39	34	5	1.16	41	0	0.94	0.76	1.15	9.55E-01
AD	Brain_Cortex	420	2626	27	21	6	1.29	26	1	1.03	0.8	1.35	9.99E-01
AD	Brain_Frontal_Cortex_BA9	330	2628	12	17	-5	0.73	21	0	0.58	0.4	0.77	9.96E-01
AD	Brain_Hippocampus	174	2751	9	9	0	1.03	12	0	0.78	0.42	1.12	8.04E-01
AD	Brain_Hypothalamus	191	2897	10	10	0	1.05	12	0	0.86	0.57	1.24	9.02E-01
AD	Brain_Nucleus_accumbens_basal_ganglia	338	2636	15	17	-2	0.89	21	0	0.72	0.5	0.97	9.95E-01
AD	Brain_Putamen_basal_ganglia	244	2605	12	12	0	0.98	16	0	0.73	0.49	0.99	9.88E-01
AD	Breast_Mammary_Tissue	676	2310	46	34	12	1.36	45	1	1.01	0.85	1.24	8.53E-01
AD	Cells_EBV-transformed_lymphocytes	464	2154	32	23	9	1.38	27	5	1.2	0.93	1.56	1.34E-01
AD	Cells_Transformed_fibroblasts	1415	1229	95	71	24	1.34	90	5	1.06	0.89	1.22	1.76E-01
AD	Colon_Sigmoid	453	2382	33	23	10	1.46	30	3	1.09	0.85	1.38	4.80E-01
AD	Colon_Transverse	736	2205	41	37	4	1.11	39	2	1.04	0.76	1.28	1.31E-02
AD	Esophagus_Gastroesophageal_Junction	437	2342	26	22	4	1.19	28	0	0.93	0.7	1.2	9.59E-01

AD	Esophagus_Mucosa	1170	1711	57	59	-2	0.97	64	0	0.89	0.77	1.05	1.00E+00
AD	Esophagus_Muscularis	1084	1621	71	54	17	1.31	70	1	1.02	0.85	1.2	2.87E-01
AD	Heart_Atrial_Appendage	629	2038	39	31	8	1.24	44	0	0.9	0.71	1.06	4.28E-01
AD	Heart_Left_Ventricle	710	1846	51	36	16	1.44	41	10	1.24	0.99	1.51	5.76E-03
AD	Liver	245	2416	17	12	5	1.39	14	3	1.25	0.8	1.72	2.38E-02
AD	Lung	1151	1793	60	58	2	1.04	81	0	0.74	0.64	0.86	1.00E+00
AD	Muscle_Skeletal	1101	1356	62	55	7	1.13	56	6	1.11	0.92	1.32	3.17E-01
AD	Nerve_Tibial	1602	1550	107	80	27	1.34	91	16	1.18	1.01	1.36	2.27E-01
AD	Ovary	230	2754	15	12	4	1.3	16	0	0.96	0.61	1.33	4.88E-01
AD	Pancreas	698	2020	51	35	16	1.46	42	9	1.22	0.95	1.48	7.20E-04
AD	Pituitary	310	2947	17	16	2	1.1	21	0	0.8	0.61	1.07	1.00E+00
AD	Prostate	213	2957	10	11	-1	0.94	12	0	0.8	0.36	1.13	3.69E-01
AD	Skin_Not_Sun_Exposed_Suprapubic	833	2022	42	42	0	1.01	50	0	0.84	0.71	1.01	1.00E+00
AD	Skin_Sun_Exposed_Lower_leg	1327	1609	93	66	27	1.4	84	9	1.11	0.95	1.28	1.98E-01
AD	Small_Intestine_Terminal_Ileum	177	3071	10	9	1	1.13	10	0	1.03	0.52	1.47	6.07E-02
AD	Spleen	410	2632	32	21	12	1.56	26	6	1.22	0.86	1.55	1.08E-01
AD	Stomach	551	2257	31	28	3	1.13	30	1	1.04	0.8	1.36	9.73E-01
AD	Testis	1340	2676	84	67	17	1.25	74	10	1.13	0.96	1.33	2.36E-01
AD	Thyroid	1523	1549	97	76	21	1.27	88	9	1.11	0.96	1.31	7.86E-01
AD	Uterus	125	2901	7	6	1	1.12	8	0	0.93	0.37	1.3	1.72E-01
AD	Vagina	120	2999	9	6	3	1.5	8	1	1.14	0.63	1.87	6.08E-01
AD	Whole_Blood	1066	1314	74	53	21	1.39	62	12	1.2	1	1.44	4.66E-01

Table S3. (A) Top 10 ranked genes in 14 brain tissues of AD based on Sherlock analysis

Rank	Brain_Amygdala	Brain_Anterior_cingulate_cortex_BA24	Brain_Caudate_basal_ganglia	Brain_Cerebellar_Hemisphere	Brain_Cerebellum	Brain_Cortex	Brain_Frontal_Cortex_BA9	Brain_Hippocampus	Brain_Hypothalamus	Brain_Nucleus_accumbens_basal_ganglia	Brain_Putamen_basal_ganglia	Brain_Spinal_cord_cervical_c1	Brain_Substantia_nigra	Pituitary
1	NKAIN1	TDRD6	PDLIM5	FABP3	BPTF	CHIT1	TBC1D2	HLA-C	SIGLEC14	MCM6	RPS3	SIGLEC14	SIGLEC14	FABP3
2	SIGLEC14	SLC1A2	SIGLEC14	TBC1D2	FABP3	SIGLEC14	SIGLEC14	SIGLEC14	HLA-C	TBC1D2	KLC3	RASGRP3	TDRD6	SIGLEC14
3	TDRD6	POLE	KIAA0040	MOB3B	CNTN6	TBC1D2	CHIT1	OPA3	TDRD6	TDRD6	HLA-C	SNX16	SLC25A27	RASGRP3
4	SLC25A27	TEX29	MPST	BPTF	SIGLEC14	ADAMTS13	SLC12A8	TDRD6	SLC25A27	POLE	TEX29	CYP21A2	LDHC	NEB
5	MGMT	HLA-C	KLC3	HLA-C	MCM6	HLA-C	TDRD6	SLC25A27	CYP21A2	SLC25A27	IKBKE	SLC25A27	LINC02145	SH3D21
6	TIPIN	DNMT3B	ACAT1	SIGLEC14	PTX3	SH3D21	HLA-C	GGH	INVS	CTNS	TDRD6	OAF	TIPIN	MAP3K3
7	CTNS	SLC25A27	HLA-C	MICA	MOB3B	ACAT1	POLE	CCDC167	LCLAT1	LDHC	MTHFSD	MGMT	GABRA4	IKBKE
8	LDHC	IFNAR1	IKBKE	DAGLB	PEX2	IKBKE	SH3D21	TIPIN	CTNS	C3orf20	SLC25A27	TIPIN	FAT1	TDRD6
9	LAT2	GGH	POLE	TDRD6	TBC1D2	TDRD6	RAB37	MGMT	TIPIN	TIPIN	SPARCL1	HLA-DRB5	PRKXP1	SLC25A27
10	B3GAT3	TIPIN	SH3D21	POLE	ATG7	POLE	SLC25A27	DPP10	LEPROT	ANXA6	FTCD	BTN2A1	MGMT	CCDC38

Table S3. (B) Top 10 ranked genes in 14 brain tissues of ND based on Sherlock analysis

Rank	Brain_Amygdala	Brain_Anterior_cingulate_cortex_BA24	Brain_Caudate_basal_ganglia	Brain_Cerebellar_Hemisphere	Brain_Cerebellum	Brain_Cortex	Brain_Frontal_Cortex_BA9	Brain_Hippocampus	Brain_Hypothalamus	Brain_Nucleus_accumbens_basal_ganglia	Brain_Putamen_basal_ganglia	Brain_Spinal_cord_cervical_c1	Brain_Substantia_nigra	Pituitary
1	CHRNA5	CHRNA5	ADAMTS7	CHRNA5	IGDCC4	NAQ	FAM86JP	CYP2A7	CHRNA5	CYP2A7	CDH23	ANXA13	SYN3	LRP1
2	PRKXP1	ADAMTS7	CDH23	IGDCC4	CHRNA5	ADAM17	PRKXP1	CHRNA5	CYP2A7	PSMA4	PSMA4	PRKXP1	PRKXP1	PRKXP1
3	WDR18	FAM86JP	PRKXP1	GRK4	ADAM17	FAM86JP	TFAM	F11	ADAM17	TFAM	PRKXP1	CYP2D6	TSNARE1	TFAM
4	FRMD4B	TPMT	ADAM17	PRKXP1	GRK4	PRKXP1	AK5	PRKXP1	PRKXP1	PRKXP1	F11	ATIC	LAYN	GRK4
5	FAM86JP	PDZD7	LINC00839	CYP2D6	PRKXP1	GRK4	PFAS	CYP2D6	WDR18	PHACTR1	LAYN	WDR18	PDE2A	RAD52
6	ATIC	PRKXP1	CYP2D6	FAM86JP	PTK2B	HEY2	CYP2D6	WDR18	NCOR2	CYP2D6	ANKRD18CP	PIGG	WDR18	CYP2D6
7	MC1R	GNAQ	GSAP	CNOT6L	LAYN	PHACTR1	RIPK1	LINC00839	TSNARE1	GRK4	WDR18	PDE2A	GTF2H2	GPR12
8	LAT2	CYP2D6	FCGBP	GPR12	CYP2D6	BTBD9	WDR18	TRPM2	SPTBN5	WDR18	ATG14	PMS2	HAPLN3	WDR18
9	CYP2D6	WDR18	WDR18	DNAJA4	FCGBP	F11	TPM1	ATG14	GTF2H2	FFAR4	RBP4	HPCAL1	GNAL	ATG16L1
10	PDZD8	NDRG4	COX11	TFAM	CHRNA2	TFAM	ATIC	HAPLN3	EPHX2	ATG14	FAM86JP	S100A10	STK31	TXN

Table S4. (A) Top 10 ranked genes in 14 brain tissues of AD based on MetaXcan analysis

Rank	Brain_Amygdala	Brain_Anterior_cingulate_cortex_BA24	Brain_Caudate_basal_ganglia	Brain_Cerebellar_Hemisphere	Brain_Cerebellum	Brain_Cortex	Brain_Frontal_Cortex_BA9	Brain_Hippocampus	Brain_Hypothalamus	Brain_Nucleus_accumbens_basal_ganglia	Brain_Putamen_basal_ganglia	Brain_Spinal_cord_cervical_c1	Brain_Substantia_nigra	Pituitary
1	ZFP69	RPL13AP5	RABGAP1L	C7orf63	ZFYVE1	HCG22	RANBP17	VN1R85P	UBXN4	FBXO21	FAM13C	FABP6	COX5A	AC011816.1
2	RP11-268J15.5	CTC-457E21.3	RP11-889L3.1	TTC28	RPS6	FGFR3	RP11-420K14.3	TC2N	HSPD1P6	HCG22	GGT1	PRDM16	TAC1	CTC-457E21.3
3	HCG22	CALR	KLF6	GNG2	TNFSF18	AC068490.2	SEZ6L2	TPMT	RBM48	SKI	ACTG1P3	ACAD8	EFCAB11	DPCD
4	PIGN	CHIT1	RPL34P34	AARS2	ANKK1	CD47	HCG22	SGCB	EFCAB11	CHCHD6	RP11-163O19.3	RP11-38M8.1	TNFSF4	PDZD7
5	RP4-789D17.5	SLC16A3	RP11-57B24.1	BMS1	ANKRA2	RFWD2	RP11-79E3.2	ROPN1B	RIMS4	C11orf54	NFAM1	TLR6	MTRNR2L12	ZNF300P1
6	NKPD1	RP11-268J15.5	PGLS	ZNF300P1	EFCAB11	CTD-3092A11.1	ZNF300P1	LINC00115	HDAC2	SNHG15	RP11-535M15.1	AC079776.2	RP11-77P16.4	RP11-268J15.5
7	MXD1	TCEA3	RP11-420K14.3	KCNAB1	ACAA2	GPR161	TARS2	CRABP1	GPR141	RP11-115H18.1	P2RX6	ZFP69	NOB1	MYO5C
8	HMMR	B3GALT6	ZNF626	MOB3B	VPS52	TOMM70A	AC002055.4	RP11-268J15.5	KRT8P26	C17orf97	LATS1	PMS2	HCG22	GTF2H4
9	EIF4E3	TRIM73	RP11-268J15.5	AC093627.10	RP5-1021I20.2	AL592284.1	SH3GLB1	B3GALT4	HSPBP1	AC007000.12	SAMD9L	ZNF649	LCP2	FABP3
10	ACSS2	RPS4XP14	GLB1L	CHTF18	WDR31	CARD11	RP11-1212A22.1	FAM86DP	OXER1	ZNF483	NEIL2	ATG14	RP11-353N4.5	ODF3B

Table S4. (B) Top 10 ranked genes in 14 brain tissues of ND based on MetaXcan analysis

Rank	Brain_Amygdala	Brain_Anterior_cingulate_cortex_BA24	Brain_Caudate_basal_ganglia	Brain_Cerebellar_Hemisphere	Brain_Cerebellum	Brain_Cortex	Brain_Frontal_Cortex_BA9	Brain_Hippocampus	Brain_Hypothalamus	Brain_Nucleus_accumbens_basal_ganglia	Brain_Putamen_basal_ganglia	Brain_Spinal_cord_cervical_c1	Brain_Substantia_nigra	Pituitary
1	CTD-2541M15.4	ZMIZ2	ADAMTS7	HSD17B8	PTCD3	ATF7IP2	SPTBN5	YWHAZP5	CRABP1	CALM2P2	PSMA4	CHRNA3	PSMA4	CHRNA5
2	STAG3L1	ADAMTS7	RSBN1L-AS1	CTD-2162K18.3	ZNF181	RP11-420K14.3	JMJD7	PRKXP1	CYP2A7	HSPB2	RP11-526I2.5	CTSH	CHRNA5	CYP2B7P1
3	TMX3	RSBN1L-AS1	ADAM17	SENP6	CHRNA5	PRKXP1	GTF2IP1	RP11-480I12.5	RP11-526I2.5	PSMA4	ZNF181	CHRNA5	CYP2A7	RP11-299H22.6
4	USP36	GNAQ	OSBPL11	PDIA2	FEZ2	RNF180	PRKXP1	KIAA1429	PRKXP1	CTSV	PRKXP1	RP11-420K14.3	RP11-661A12.14	HCG17
5	RFT1	FEZ2	RP11-526I2.5	RSBN1L-AS1	RP11-420K14.3	ADAM17	RPAP1	RP11-526I2.5	NR1D1	HMG20A	CHRNA5	DUSP14	RP11-17L5.4	PRKXP1
6	WDR18	HDDC2	RP13-977J11.2	CHRNA5	RSBN1L-AS1	AC110754.3	RP11-420K14.3	RP11-420K14.3	RSBN1L-AS1	CRLF1	PSMC5	RSBN1L-AS1	RP11-526I2.5	NSUN6
7	RP11-497H16.9	ZNF181	PRKXP1	RP11-420K14.3	RP11-526I2.5	RP11-111K18.2	AMFR	LINC00839	ASS1P2	RP11-497H16.2	SHARPIN	PSMB9	ZBTB8A	AACSP1
8	C5orf56	CHRNA5	DZIP1L	FEZ2	PRKXP1	OVOL1	CHRNA5	FTSJ3	LINC00115	CNOT6L	RP11-561C5.5	ASAP2	STAG3L1	MRPL43
9	PCDHGB2	BBS2	CHRNA5	AACSP1	ADAM17	ZNF181	RP11-665C16.6	GNE	RP11-480I12.7	CHRNA5	GTF2H2	PIWIL2	CCDC88A	ZMIZ2
10	WIPF1	RP11-420K14.3	SPTBN5	RPL5P11	RP11-490M8.1	GTF2F2P2	STRA13	ZNF517	MRPL43	AC078941.1	PLA2G4B	DDX42	NBPF3	RP11-420K14.3
